# Investigation on mechanical properties and corrosion resistance of Ti-modified AA5083 aluminum alloy for aerospace and automotive applications

**DOI:** 10.1038/s41598-023-38510-1

**Published:** 2023-07-17

**Authors:** Abdullah A. Alghannam, Mahmoud S. Soliman, Asiful H. Seikh, Ibrahim A. Alnaser, Ahmed Fouly, Jabair A. Mohammed, Sameh A. Ragab, Hany S. Abdo

**Affiliations:** 1 Mechanical Engineering Department, College of Engineering, King Saud University, 11421 Riyadh, Saudi Arabia; 2 Centre of Excellence for Research in Engineering Materials (CEREM), Deanship of Scientific Research, King Saud University, 11421 Riyadh, Saudi Arabia

**Keywords:** Mechanical engineering, Materials science

## Abstract

Casting of aluminum with different concentration of alloying elements such as Mg, Mn (similar to that in AA5083) with additional percentages of 0.1, 0.2 and 0.3% Ti, are carried out using graphite crucible. The as-cast microstructure is modified by hot rolling to a thickness of ~ 2 mm. Mechanical and metallurgical and characterization of heat-treated thin sheets are carried out using tensile testing, hardness measurement, metallography, image analysis and optical microscope. By increasing the Ti content, the results show grain refinement and increase in the formation of Al3Ti which reflected positively on the mechanical properties. Specifically, Ultimate tensile strength is increased from 260 MPa (0 wt% Ti) to 345 MPa (0.3 wt% Ti) when using water quenching, 32.6% improvement for air cooling, and 23.3% for furnace cooling. Electrochemical corrosion behavior of heat-treated water quenched, air cooled and furnace cooled samples were tested in 3.5% NaCl solution. The results show that the heat-treated alloys have very good resistance against corrosion, while by increasing the Ti content, the corrosion rate increases due to the grain refinement phenomena.

## Introduction

Aluminum alloys have been widely used as structural materials in the aircraft industry for several decades^1^. This is because most aluminum alloys have a better strength-to-weight ratio compared to other structural materials, such as high-strength steel alloys^2,3^. This is mainly due to the low density of aluminum (Al), which is about one-third that of steel alloys^4–7^. Among the many alloying elements that can be added to aluminum (Al), the most widely used ones are magnesium (Mg), copper (Cu), silicon (Si), zinc (Zn), and manganese (Mn). The types and contents of the added alloying elements determine the different classes of cast and wrought aluminum alloys^8–11^. The alloying elements of Mg and Cu are usually added to make solid solution strengthening or precipitation hardening e.g. 5xxx and 2xxx series, respectively of Al alloys. Addition of Ti as grain refiner, in the form of Al–5Ti–B, is mainly due to formation of fine particles of Al3Ti intermetallic compound. The increase in strength is due to double action of Al3Ti particles. It will work as nucleating agent to provide sites for heterogeneous nucleation and refinement of grain size. In addition, these particles will increase the strain hardening by working as barrier for dislocation motion.

In particular, the use of magnesium and manganese, along with other additives, constitutes the most important aluminum alloy known for its distinctive properties (Al–Mg–Mn alloys), which are classified under the 5000 series^12^. The Al–Mg–Mn alloys are known for their excellent mechanical properties, such as high strength, high thermal stability at elevated temperatures, good corrosion resistance, and good ductility^1,13–16^. Titanium is also a well-known element in improving the mechanical properties of aluminum alloys when added, according to many studies^17^, due to the formation of a new precipitation phase called Al3Ti (titanium aluminide). A recent welding study of AA5083 reported that the presence of Al3Ti is the main reason for the enhanced hardening process in AA5083 +Ti alloys^12^. However, the effects of various processing parameters and microstructure. For example, the combined effects of compositions (e.g. weight percentage of Ti element), and Al_3_Ti microscopic features (e.g. morphology, density, size, and distribution) on the hardening process clearly need more investigations in these alloys^18^.

Murayama et al.^19^ found the effect of adding small amount of Ti to form Al_3_Ti particles, which act as refining agent of grain size during solidification and as strengthening particle that act as obstacle to dislocation motion during plastic deformation. Refining grains by changing grain size using different techniques is a preferred method to improve material properties^20–23^. There are many ways to reduce grain size, either by adding grain refining agent or severe plastic deformation^24,25^. A study was conducted in which the controlled quantities of Tibor TM (containing Ti and B by 5: 1 ratio) were included in the molten series of AA 5083 by pre-casting deposit under different laser welding conditions. The results show that, despite the high cooling rate and great melt overheating, the laser weld could be grain refined to a mean grain size of 22 μm^26^. The analysis has shown that Al3Ti was confirmed as the nucleus of parallel grains; fine particles of Al_3_Ti are serving as nucleanat substrates during solidification^18,27^ promoting heterogenous nucleation and fine grain size. This is why the research idea started so that we focus on titanium addition in the form Al–5Ti–B to form Al3Ti particles, which is formed in the shape of small particles that strengthen the alloy and refine grain size. A significant improvement after the addition of titanium in the few percentages (0.1, 0.2 and 0.3) could be achieved. To the best of our knowledge, there is no previous research of adding Ti as grain refiner during the melting of AA5083.

Fanyong et al.^28^ performed a duplex surface treatment via depositing Ti film followed by plasma nitriding on 5083 Al alloy to produce nitride/intermetallic layers with enhanced mechanical properties. The results show a remarkable increase in surface hardness (up to 900 HV) and the wear resistance of 5083 Al alloy was improved significantly. While Zhang et al.^29^ have developed a novel method to fabricate a multiphase coating containing both nitride and intermetallics on GB-5083 Al alloy, with the aim to harden its surface properties. The method was involved with two steps, i.e., depositing pure Ti film on GB-5083 Al substrate and post plasma nitriding the Ti coated substrate. The surface hardness of the multiphase coating reached to 600 HV, five times harder than 5083 Al alloy.

Patel et al.^30^ studied the effect of ultrasonic stirring on changes in microstructure and mechanical properties of Al 5083 and Al 5083-TiC composites. They concluded that Al3Ti with the properties of high rigidity and hardness can be used as a good reinforcement if its morphology can be controlled. Moreover, Kishore et al.^31^ were analyzed and optimized the corrosion rate parameters for the immersion corrosion test to minimize the corrosion rate for the aluminum alloy AA5083 reinforced by Titanium Diboride. The optimized combination of parameters for reduced wear rate was 12% reinforcement, 3% of HCL and 144 h of Immersion time.

Cold working generally reduces the corrosion resistance of the magnesium-alloyed Al grades, as the β-Al_3_Mg_2_ phase may precipitate on grain boundaries and dislocations, increasing susceptibility to stress corrosion cracking^32^. Inclusions, impurities, pores, vacancies, dislocation walls and grain boundaries may generate galvanic cells in 5083 alloy. Cored structures promote galvanic interaction and point defects are usually more anodic than the surroundings^33,34^. Intermetallic phases, such as Al_3_Mg_2_, Al_3_Mg_5_ and Mg_2_Si, are anodic with respect to the 5083-alloy matrix, and promote rapid localized attack through galvanic interaction. Less electronegative intermetallic phases, such as Al_3_Fe and Al_6_Mn, are cathodic with respect to the 5083-aluminum matrix, leading to preferential dissolution of the alloy matrix^34,35^.

Considering the fact that no study has been found in the literature on the corrosion and mechanical properties of hot rolled Al–Mg–Mn–Ti alloys, the current study aims to investigate the effect of Ti addition by different percentages (0.0, 0.1, 0.2 and 0.3) wt% on the mechanical and electrochemical properties of Al–Mg–Mn (AA5083) alloy.

## Experimental procedures

### Materials

Four aluminum alloys of AA5083, with different Ti percentages, were prepared. The first alloy is free of titanium, and titanium was added to the other three alloys, as 0.1, 0.2 and 0.3% as shown in Table1.

**Table 1:** Planned chemical composition of the studied alloys.

Alloy wt%	Al	Mg	Mn	Fe	Si	Cr	Cu	Ti
Reference	Balance	4–4.9	0.4–1.00	0–0.4	0–0.4	0.05–0.25	0–0.1	0.05–0.25
Sample 1	Balance	4.5	0.6	0.2	0.2	0.1	0.05	0.0
Sample 2	Balance	4.5	0.6	0.2	0.2	0.1	0.05	0.1
Sample 3	Balance	4.5	0.6	0.2	0.2	0.1	0.05	0.2
Sample 4	Balance	4.5	0.6	0.2	0.2	0.1	0.05	0.3

The start was by preparing the required amounts pure metals of Al, Mg and other elements, melting them, then proceed with casting in order to form alloys, as shown in Table 1. The manufacturing process for the selected compositions started by raising the raw aluminum to a temperature of 730 °C, then 5083 alloy elements were added, and the process was repeated three times to add different contents of titanium, which is the subject of the study. The weight of each alloy was calculated to provide the desired chemical composition as described in Table 1.

### Procedures

The melting process was carried out carefully to ensure complete dissolution of the elements in the alloy. It began by placing the Al in a graphite crucible in the furnace and raising the temperature to 730 °C until Al is melted. After that, magnesium was added with mixing and left for 25 min, then addition of manganese powder and kept for extra 15 min. Finally, the Ti was added by different amounts to produce different Ti content samples. Before casting begins, the steel mold is heated to a temperature of 450 °C and kept at this temperature for 3 h to avoid the influence of the temperature difference between the ingot and the mold walls, and the alloy is kept in the room until the temperature reaches the room temperature level. The casted plate has dimensions of 104 × 64 × 19 mm.

Homogenization process is carried out in a furnace at 450 °C for 24 h after the casting process to ensure a uniform distribution of all alloying elements, reduce casting defects, vacancies and pores associated with the casting and furnace cooled to room temperature, and the homogenization process may take two days, and after its completion, the outer surface of 2 mm thickness of the alloy is removed to ensure that there is no oxidation.

The hot rolling takes place after the first homogenization process and it is by inserting the ingot with dimensions ~ 15 × 60 × 100 mm into the rolling device consisting of two hot rollers at temperature of 200 °C and the samples were heated to a temperature of 450 °C for 2 h and rolled directly under the hot rolls from 15 mm thickness by 10% reduction to thickness 13.5 mm. This process of heating and rolling with a gradual decrease of 10% each time has been repeated to a final thickness of ~ 2mm. with an average speed of 1.75 m/min. And every time the sheet comes out of the rolling device, the resulting thickness is measured and directly inserted into the oven for 10 min. The temperature of 450 °C was also chosen based on the percentage of magnesium in aluminum, which is a medium percentage, such that Mg will be in solid solution.

After the rolling and homogenization process is completed to confirm the composition of the alloy material with the required alloy, a grinding machine is used to remove the oxidation layer and smooth the surface of the alloy. After that, we use the SPECTROMAX metal analyzer after adding a dedicated electrode to the aluminum alloy. And the program format selects Al–Mg alloy. Then the analysis is started, and at least the average of seven readings is taken.

During water quenching (WQ), all hot-rolled samples underwent solid solution treatment at 520 °C for 3h followed by rapid quenching in water. The process is required to form a supersaturated solid solution (SSSS) where all minor phases are dissolved in the α-aluminum matrix.

Air cooling (AC): at this stage, samples were subjected to solid solution treatment at 520 °C for a period of 3 h, and then they were removed from the oven to room temperature to be air-cooled at room temperature. Furnace cooling (FC): at this stage, some samples were subjected at 520 °C for a period of 3 h, then the heat source was closed without opening the oven until 48 h had passed, so that the cooling was gradually due to the leakage of heat from the oven to the outside.

Hardness testing: after the solid solution process, the samples were prepared for the hardness test. The preparation started with grinding the samples with 500 grit in one direction followed by grinding with 1200 grit. The Wilson Instruments (WOLPRET UH930) machine used to test the hardness. The Vickers hardness test is used at load 10 KgF for dwell time of 15 s. The result is taken as an average of 5 measurements taken in different areas of the sample.

Tensile testing: Tensile samples are produced after the heat treatment of three types, quenching, air, and furnace cooling and shaped is in accordance with ASTM E 8/E 8M-08. The tests were carried at initial strain rate of 10^−3^ s^−1^. Stress-strain curves were plotted for each category. These tensile results were used to show the effect of Ti ratio on the yield strength, ultimate tensile strength and ductility.

Optical microscopy was used to relate microstructure and mechanical properties resulting from tensile and hardness tests. Scanning with optical microscopy requires preliminary preparation of samples by mixing an appropriate amount of resin and hardener to mount the samples to be easily handled on the grinding machine. Grinding, using BUEHLER equipment, begins with lower to higher grades in the following order 60, 180, 360, 660, 900, 20000, 40,000, then polishing with 0.05 μm alumina particles. Then the etching process is with a solution of ASTM E 407-99 embossing standard No. 3 (2ml HF, 3ml HCl, 5ml HNO3 and 200ml H2O). The samples are immersed in solution for 45 s to determine the boundaries of the grains, but also allows the different phases to be distinguished with different grain brightness, shape and color. The granular and morphological structure was observed by an Olympus light microscope. Scanning electron microscopy (SEM) was performed using a JXA- 840A electron probe microanalyses (JEOL, Japan), this analysis was carried out to have a quantitative analysis of the composition by utilizing the EDS analysis. The same samples of optical microscopy which were mounted on the resin were used. The sample for the SEM observations needs to be electrically connected to the sample holder to prevent the electron beam from “charging” the sample and distorting the image. This is usually done through conductive tape.

The electrochemical tests were conducted in 3.5% NaCl solution using a three-part system comprising of the counter electrode, reference electrode, and heat-treated Al alloys chosen as the working electrode^36^. A copper wire was welded to the upper sample surface before the assembly was mounted by epoxy. The samples were then polished to obtain a smooth surface for electrochemical measurements. The electrochemical tests were carried out using a potentiostat/galvanostat (Autolab PGSTAT302N) with potential from −1500 to +1500 mV and a scan rate of 1 mV/s accompanying with Nova software.

## Results and discussions

### Chemical composition analysis

Table 2 shows the chemical analysis results released by the metal analyzer (SPECTROMAX) for all samples tested in this study. The results also indicate that the measured chemical compositions of all samples that were used in the study fall within an acceptable range for the mother alloy 5038, which is presented in the experiments section.

**Table 2:** The actual chemical composition analysis by SPECTROMAX Metal Analyzer.

Alloy wt%	Al	Mg	Mn	Fe	Si	Cr	Cu	Ti
Sample 0	Balance	4.2300	0.648	0.0499	0.0707	0.0757	0.0373	0.0156
Sample 1	Balance	4.3931	0.620	0.0505	0.0731	0.0973	0.0811	0.091
Sample 2	Balance	4.6042	0.1352	0.0450	0.0602	0.0722	0.0346	0.2224
Sample 3	Balance	4.6741	0.239	0.0239	0.0624	0.0404	0.0413	0.3498

### Microstructural observation by optical microscopy

**Figure 1:**
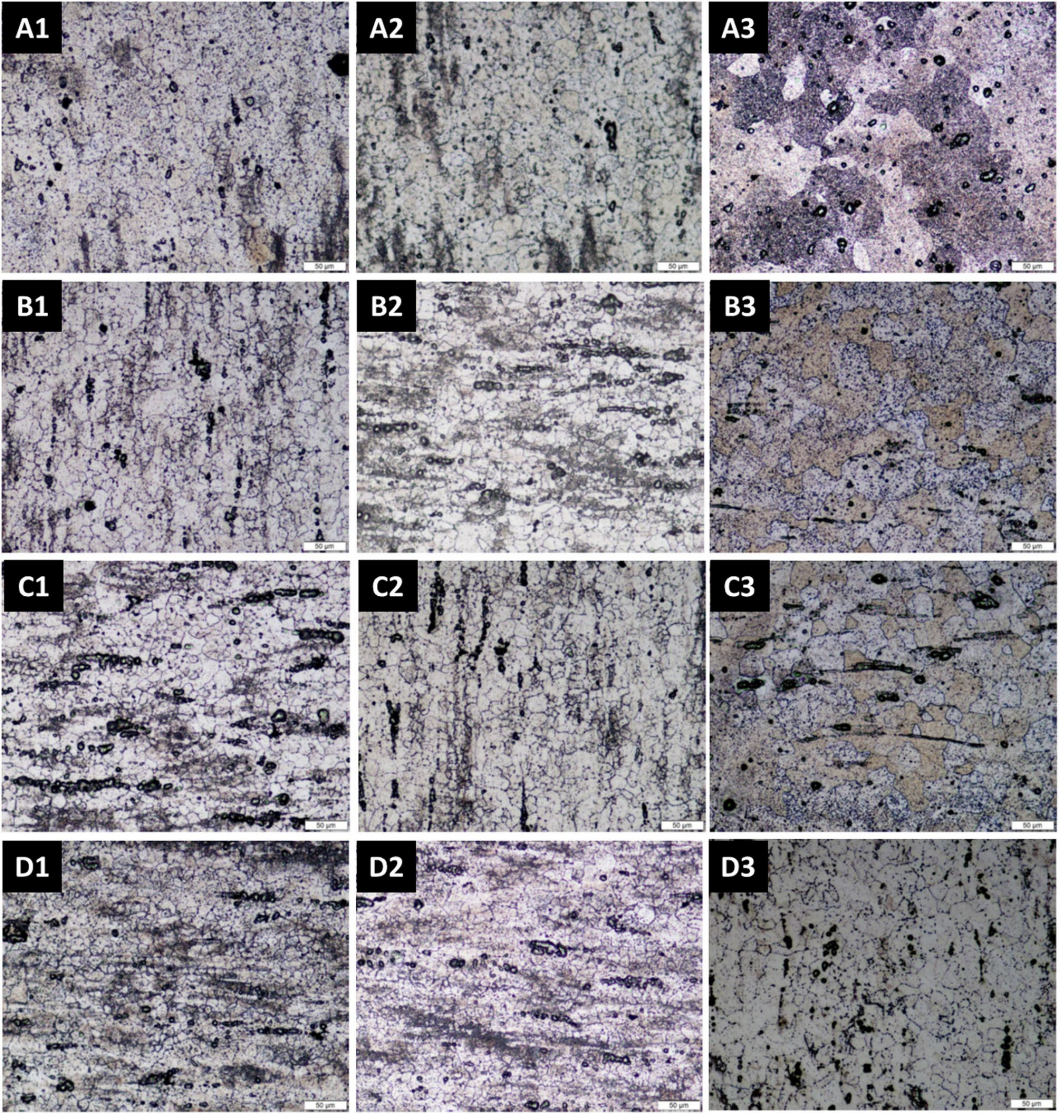
Optical micrographs of etched surfaces for (**A**) 0 wt%Ti (**B**) 0.1 wt%Ti (**C**) 0.2 wt%Ti (**D**) 0.3 wt%Ti, and (1) Quenching cooling, (2) Air cooling, (3) Furnace cooling.

The microstructure of the WQ alloy of AA5083 of 0% Ti is shown in Fig. 1a with an average grain of 15.24 μm. For WQ alloys, when 0.1% Ti is added with AA5083 alloy, the average grain size is 12.43 μm, when the Ti ratio is increased by 0.2%, the average grain size decreased to 10.26 μm, and at the high content of 0.3% Ti, the average grain size decreased to 8.72 μm, as shown in Fig. 1b. This decreases in grain size when titanium is increased and that the percentage of grain refining after adding 0.3% titanium is 43%, as shown in Fig. 1. The microstructure of the air-cooled alloy with 0 and 0.3% Ti are shown in Fig. 1c,d, respectively.

The decrease in the size of the grains, from 16.91 to 14.11, for 0.1% Ti, then 11.45 for 0.2% Ti, and then at high 0.3% Ti, to be 9.97 μm, with a grain size reduction of 41%. The microstructure of the FC alloys is shown in Fig. 1e,f for 0 wt% and 0.3 wt% Ti, respectively. The average grain size is of 19.63 μm for 0% Ti. The decrease in the size of the grains, from 19.63 to 17.08 μm, for 0.1% Ti, then to 14.40 μm for 0.2% Ti, then at high of 0.3% Ti, to be 11.94 μm, with reduction of 39%.

**Figure 2:**
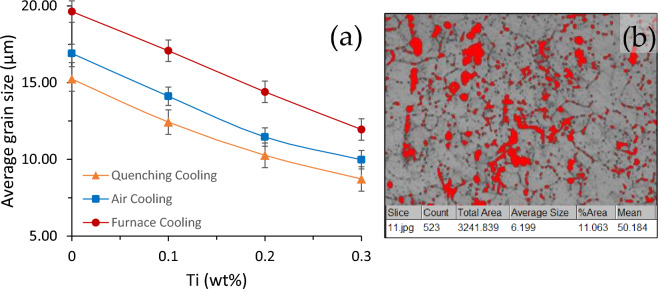
(**a**) The variation of the average grain size with the Ti content for the different cooling methods, (**b**) Average size and the volume fraction of the bead’s particles for the quenched 0.3 wt% Ti sample.

The optical micrographs, Fig. 1, clearly show that the fine structural conformation changes from a course interlocking structure of varying sizes to a fine-grained balanced microstructure due to the addition of a Ti-grain refiner. Figure 1 shows the optical micro-structures of the AA5083 aluminum alloy before and after grain refinement. It is evident from Fig. 2a that the addition of Ti led to refining the grains of AA5083 alloy grains. From Fig. 1a, it is found that the unrefined microstructure of AA5083 alloy is composed of different microstructure in grain size, heterogeneity and size uniformity which is dominated by large size. The addition of Ti to the AA5083 alloy led to changes that reduced the grain size with a homogeneous percentage as shown in Fig. 1c. It was observed that refining the grains increased the grain boundary area per unit volume. This ensures the uniform distribution of the insoluble substrates inside the matrix, which act as a primary nucleus initiation. \

### EDS analysis and density measurements

In order to clarify the effect of titanium addition on the morphological evaluation of AA5083 alloys, an energy dispersive spectroscopy (EDS) was performed for the internal structure of the samples by detecting the size, shape and composition of the precipitate in the Al matrix. The quenched alloy AA5083 with 0.3% titanium addition contained some deposits in the form of beads with an average size of 6 μm, and the volume fraction of these particles was 0.11% as analyzed by ImageJ software and presented in Fig. 2b. the chemical composition by EDS analysis for the present alloys, as shown in Table 3, is in good agreement with Spectromax analysis, Table 2. The density of the produced samples has been investigated and measured by Archimedes principal. The relative density of 0, 0.1, 0.2 and 0.3 wt% Ti alloys are 99.5, 99.4, 99.6 and 99.5 respectively.

**Table 3:** The chemical composition analysis by EDX for the quenched cooling alloys.

Unmodified AA5083 alloy		Quenched cooling AA5083 alloys		
Element	Weight%	0.1 wt% Ti	0.2 wt% Ti	0.3 wt% Ti
Al	95.52 ± 0.52	95.03 ± 0.64	95.37 ± 0.57	95.08 ± 0.77
Mg	4.28 ± 0.11	4.60 ± 0.13	4.17 ± 0.12	4.40 ± 0.14
Mn	0.16 ± 0.02	0.34 ± 0.03	0.42 ± 0.02	0.47 ± 0.03
Fe	0.04 ± 0.01	0.03 ± 0.01	0.04 ± 0.01	0.05 ± 0.01

### Mechanical properties

#### Hardness testing measurement

**Figure 3:**
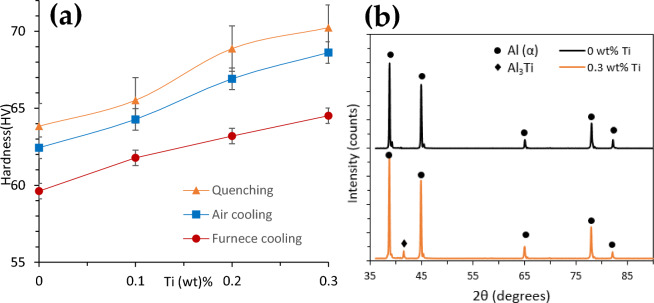
(**a**) The variation of the hardness values with the Ti content of the different cooling methods, (**b**) XRD pattern for the quenched 0.3 wt% Ti sample.

To understand the effect of alloying and pre-deformation on the hardness of cast samples, the Vickers Hardness machine was used to measure the hardness of each sample. Figure 3a shows the hardness values of unmodified and Ti modified alloys. The plots in Fig. 3a are the average values of five test specimens made from single castings. It was found that the hardness values increase with increasing Ti content, which is mainly attributed to grain refinement and Al3Ti formation as shown in the XRD pattern in Fig. 3b. The increase in hardness due to the addition of the titanium element in the Al3Ti alloy is mainly due to the formation of fine particles of Al3Ti that acts as grain refiner and results in the formation of fine grain size. The hardness increases mainly due to the increase in the grain boundary area per unit volume and in the amount of Al3Ti particles and thus, the increase in the grain boundaries and Al3Ti particles increase the barrier for dislocations motion, and the effect is reflected on the hardness values.

#### Tensile testing measurements

**Figure 4:**
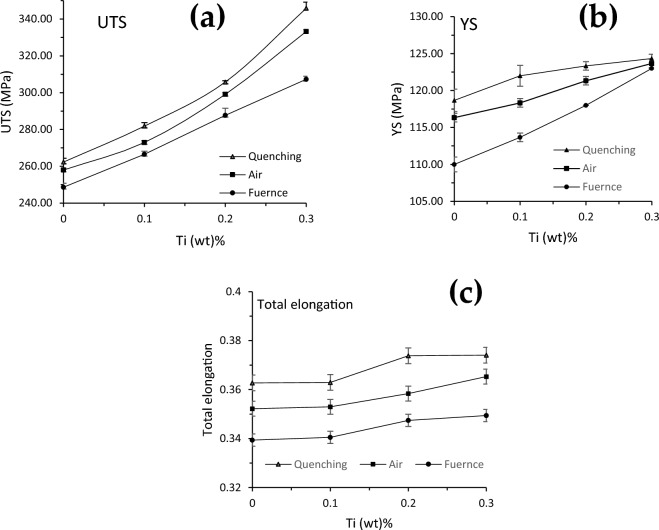
Tensile test results of AA5083 alloys as a function of Ti content after different cooling methods: (**a**) Ultimate tensile strength UTS, (**b**) Yield strength YS and (**c**) elongation%.

After, the solid solution treatment and sample cooling, the results of AA5083 alloys were compared. The tensile samples were tested to determine the effect of Ti addition on the mechanical properties of AA5083 alloys. The results showed that adding Ti has increased the ultimate tensile strength (UTS), yield stress (YS) and elongation% by 22%, 5.6% and 5%, respectively, in the AC process. From Fig. 4a, it can be observed that the UTS is gradually increasing with an increase in Ti content from 257 to 333 MPa for 0 to 0.3% Ti, approximately 22% increase. Along with a reasonable increase in yield and elongation by 5.6 and 5%, respectively. The elongation can be ignored since it is a small value compared by the improvement in the ultimate strength. Similarly, the FC samples have shown a considerable increase in UTS, YS and elongation by 19%, 10.5% and 3.5%, respectively. UTS, YS and total elongation of AA5083 and AA5083+xTi (x=0.1% - 0.3%) alloys are shown in Fig. 4. Figure 4. shows comparison between the tensile properties of all samples. It is clear that small grain size gives better mechanical properties.

### Corrosion Study

**Figure 5:**
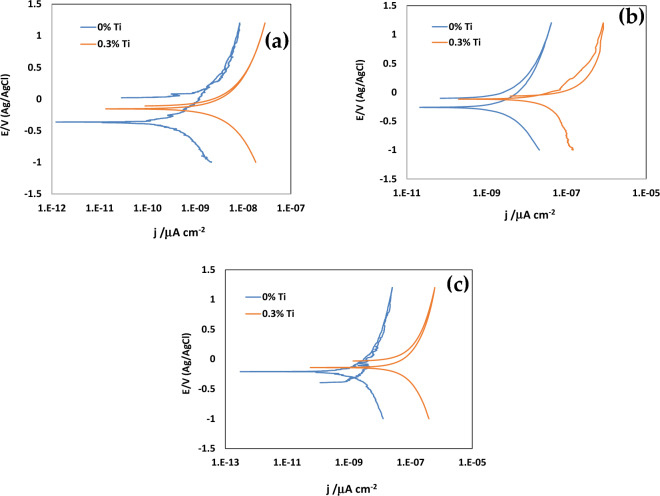
Cyclic potentiodynamic polarization (CPP) curves for (**a**) WQ; (**b**) AC and (**c**) FC samples after 1 h immersion in 3.5% NaCl solution.

Cyclic Potentiodynamic Polarization (CPP) was used to examine the corrosion behavior of different heat treated Al alloys by immersing them in 3.5% NaCl solution for 1 h^37^. The polarization results of the different heat-treated alloys are shown in Fig. 5. Electrochemical parameters such as corrosion current density, j_corr_ (mA/cm^2^) and corrosion potential E_corr_, V (Ag/AgCl) were calculated after fitting the polarization curves in Tafel’s regions; results are shown in Table 4.

**Table 4:** Electrochemical parameters obtained for WC, FC and AC samples after 1 h immersion in 3.5% NaCl solution.

Percent	WC		AC		FC	
	Ecorr, V (Ag/AgCl)	Jcorr, μA/cm^2^	Ecorr, (Ag/AgCl)	Jcorr, μA/cm^2^	Ecorr, V (Ag/AgCl)	Jcorr, μA/cm^2^
0%Ti	− 0.365 ± 0.024	0.0012 ± 0.001	− 0.325 ± 0.026	0.0006 ± 0.000	− 0.210 ± 0.019	0.0002 ± 0.000
0.3%Ti	− 0.180 ± 0.010	0.0180 ± 0.005	− 0.155 ± 0.017	0.0120 ± 0.003	− 0.122 ± 0.011	0.0042 ± 0.002

The polarization curves and the data obtained clearly show that the corrosion current (j_corr_ value) for all the heat treated samples are very low i.e. the produced alloy is very much corrosion resistance^38^. Though with the increasing %Ti the corrosion rate for all the differently heat-treated samples are increases. The corrosion potential (E_corr_ value) also increased with increasing Ti. The grain refinement of this alloy increased with increasing Ti addition as observed before. It is also seen that the jcorr value for FC sample is lower than the samples of AC and WC. That means the for WC sample the corrosion rate is higher than that of AC and FC. This is good agreement with the grain size results found before (Fig. 2). When grain refinement occurs, it provides more sites to initiate the corrosion (i.e., increases the corrosion rate, which decreases the overall corrosion resistance property). Grain refinement provides more areas for corrosion in two ways: (1) inside the grain boundary and (2) at the grain boundary. The results clearly indicate that with more Ti percentage the tendency of corrosion rate increase in the Al alloy, due to grain refinement^39^.

## Conclusions

The current study investigates the impact of adding Ti as a grain refiner on the fine grain size, mechanical properties, electrochemical properties, and corrosion behavior of AA5083 alloys. The microstructure and mechanical properties of AA5083 alloys were analyzed in relation to the cooling method used in the process. The results showed that incorporating Ti into AA5083 alloy decreased the average grain size from 15.24 to 9.72 µm when employing water quenching. Meanwhile, the grain size reduction ranged from 16.91 to 9.97 µm with air cooling, and from 19.63 to 11.94 µm when using furnace cooling. Furthermore, with the increase of Ti fraction from 0 to 0.3, the ultimate tensile strength rose by 32.7% with water quenching, 29% with air cooling, and 23.3% with furnace cooling. The enhancement in the AA5083 alloy strength can be attributed to the interaction between the added Ti particles and metal dislocations, which impedes the movement of dislocations and makes it more difficult for the metal to deform. Moreover, the addition of Ti not only impedes dislocation movement, but also changes the initial phase morphology by refining the grains through the formation of Al3Ti and Ti separation, resulting in a finer grain structure. Eventually, water-quenched alloy has lower corrosion resistance compared to air-cooled and furnace-cooled alloys due to its finer grain size. Additionally, the corrosion resistance of the 0.3%Ti alloy, which has undergone significant grain refinement, is inferior to that of the free Ti alloy.

## Data Availability

The data used to support the findings of this study are available from the corresponding author upon request.
